# A Role of Inflammation and Immunity in Essential Hypertension—Modeled and Analyzed Using Petri Nets

**DOI:** 10.3390/ijms21093348

**Published:** 2020-05-09

**Authors:** Dorota Formanowicz, Agnieszka Rybarczyk, Marcin Radom, Piotr Formanowicz

**Affiliations:** 1 Department of Clinical Biochemistry and Laboratory Medicine, Poznan University of Medical Sciences, 60-806 Poznan, Poland; doforman@ump.edu.pl; 2 Institute of Computing Science, Poznan University of Technology, 60-965 Poznan, Poland; Agnieszka.Rybarczyk@cs.put.poznan.pl (A.R.); Marcin.Radom@cs.put.poznan.pl (M.R.); 3 Institute of Bioorganic Chemistry, Polish Academy of Sciences, 61-704 Poznan, Poland

**Keywords:** hypertension, inflammation, immunological phenomena, mathematical modeling, Petri nets, t-invariants

## Abstract

Recent studies have shown that the innate and adaptive immune system, together with low-grade inflammation, may play an important role in essential hypertension. In this work, to verify the importance of selected factors for the development of essential hypertension, we created a Petri net-based model and analyzed it. The analysis was based mainly on t-invariants, knockouts of selected fragments of the net and its simulations. The blockade of the renin-angiotensin (RAA) system revealed that the most significant effect on the emergence of essential hypertension has RAA activation. This blockade affects: (1) the formation of angiotensin II, (2) inflammatory process (by influencing C-reactive protein (CRP)), (3) the initiation of blood coagulation, (4) bradykinin generation via the kallikrein-kinin system, (5) activation of lymphocytes in hypertension, (6) the participation of TNF alpha in the activation of the acute phase response, and (7) activation of NADPH oxidase—a key enzyme of oxidative stress. On the other hand, we found that the blockade of the activation of the RAA system may not eliminate hypertension that can occur due to disturbances associated with the osmotically independent binding of Na in the interstitium. Moreover, we revealed that inflammation alone is not enough to trigger primary hypertension, but it can coexist with it. We believe that our research may contribute to a better understanding of the pathology of hypertension. It can help identify potential subprocesses, which blocking will allow better control of essential hypertension.

## 1. Introduction

Although hypertension is a common disorder, there is still uncertainty about the underlying mechanisms. Hence only a small number of patients are adequately treated, avoiding cardiovascular complications. In almost 95% of patients with hypertension, the cause of this disease is unknown, and they are referred to suffer from essential hypertension. Currently, there is more and more evidence that hypertension is not only hemodynamic disorder but also a very complex disease that includes numerous abnormalities, such as inflammatory process, activation of the immune system, metabolic problems, abnormal fat distribution and over-activity of the sympathetic nervous system, becoming the probable cause of treatment failure in many patients. This complexity of hypertension was already noticed by Dr. Guyton over 50 years ago. In 1961 he developed a computational model predicting how the kidney is the center-point of long-term blood pressure control. Then, together with Coleman, he proposed the linkage between blood pressure and natriuresis, which maintains the balance of sodium and extracellular volume in the body [1,2]. These pioneers’ research, far ahead of their times, laid the groundwork for intensive research on the mechanisms of arterial blood pressure regulation.

To the best of our knowledge, there is no research on essential hypertension which, in order to capture new properties, would simultaneously take into account many of the fundamental processes underlying the disease and analyze their interrelations in a single project. In our study, to accomplish this, we conducted in silico experiments using a systems approach based on Petri net theory. In general, the systems approach used to model complex biological systems involves a wide range of different techniques, such as ordinary differential equations (ODEs), Boolean networks (BNs), linear programming (LP), or agent-based models (ABMs). ODE-based methods that are frequently applied for this purpose, despite their expressive power, also have some limitations. They result from the necessity to determine the exact values of some parameters corresponding to the quantitative properties of the modeled system. The values of these parameters are often difficult to obtain in practice, and this makes the construction of the ODE model a difficult and sometimes impossible task. Models based on ODEs have also proved suitable for modeling continuous kinetic changes in small networks, see [3,4]. Logical modeling techniques, ABM, and LP also suffer from some limitations that have been thoroughly discussed, for example in [3,4,5]. In contrast to the methods mentioned above, Petri nets provide an intuitive graphical representation that is of great importance during the development phases of the biological system and gives the possibility to simulate system behavior. Moreover, Petri nets are mathematical objects, and thus, can be analyzed using strict mathematical methods.

Furthermore, there are several different extensions of the classical (qualitative) Petri nets, which make it possible to incorporate information of different types in models based on them, increase their precision and allow to meet the modeling and analysis needs of diverse, complex dynamical systems. Among these extensions are stochastic Petri nets (SPNs) [6], used for instance to study complex disease comorbidities in autism and inflammatory bowel diseases (see [7]), to demonstrate the significance of IL-10 in the inflammatory process (see [8]) or to study interferon regulatory factors genes and T cells activation (see [9]). Additionally, Bolton with his co-authors [10] have proposed a new type of net, i.e., Fuzzy Continuous Petri net, which allows using fuzzy firing rates for transitions. However, reliable reaction data are, in most cases, not accessible in the literature and the structure of the net itself determines many fundamental properties of the analyzed system and still results in many interesting and spectacular discoveries.

In our study, using the classic Petri nets used by other researchers, we investigated the factors and mechanisms underlying primary hypertension, whose role in the light of recent research seems to be crucial. In the beginning, we created a model in which we took into account both the role of the RAA system, whose significance is undisputed, as well as on new scientific discoveries, such as the significance of neoantigens [11], the existence of an additional salt buffering mechanism [12], the role of oxidative stress, inflammation and endothelial dysfunction [13]. To understand the importance of studied factors/mechanisms, we blocked some of them during the knockout analyzes and then checked how the system (representing the human body affected by hypertension) works without them. It allowed assessing the importance of particular factors.

The article has four main sections. Firstly, the problem of the complexity of primary hypertension with particular emphasis on the new scientific trends is shown. The next part briefly discusses the methods used in this study (there are two main sub-sections here, the first is the part presenting Petri net, the second is a description of the hypertension phenomenon, enriched with a block diagram enabling understanding the analyzed issue. Then, there is a section of results and discussion, when the model and its formal analysis are presented. Moreover, six scenarios are analyzed and discussed here. Each of them includes questions and answers, accompanied by figures and tables that facilitate understanding of the analyzes. The last section contains conclusions.

## 2. Methods

### 2.1. Petri Nets

**Figure 1:**
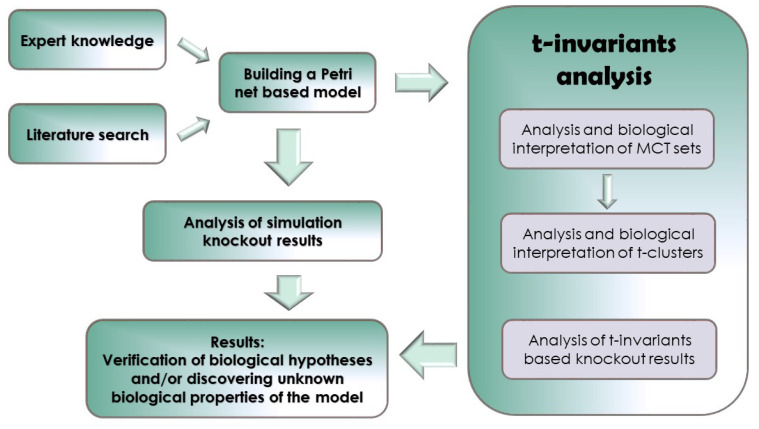
The general working scheme describing the steps that should be taken in order to create model, conduct the analysis and obtain results.

In the beginning, we would like to present how Petri nets are used in bio-medical research. What is their place and on what basis the models are created and then analyzed. Figure 1 shows the block diagram of this method.

Petri nets are mathematical objects whose structure is a directed bipartite graph. Graph G=(V,A) of this type is composed of two subsets of vertices $V_{1}$ and $V_{2}$ such that $V=V_{1}\cup V_{2}$, $V_{1}\cap V_{2}=\emptyset$ and $\forall_{(v_{x},v_{y})\in A}\left(v_{x}\in V_{1}\wedge v_{y}\in V_{2}\right)\vee \left(v_{x}\in V_{2}\wedge v_{y}\in V_{1}\right)$ (i.e., there is no arc in the graph connecting two vertices being elements of the same subset). In a Petri net, vertices belonging to one of these subsets are called places, while vertices being elements of the second subset are called transitions. (From this it follows that no two places nor two transitions can be connected by an arc.) For transition $t_{j}$ place $p_{i}$ is called its pre-place if there exists arc $\left(p_{i},t_{j}\right)$, i.e., if place $p_{i}$ is an immediate predecessor of transition $t_{j}$. Analogously, place $p_{k}$ is called post-place of transition $t_{j}$ if there exists arc $\left(t_{j},p_{k}\right)$, i.e., if place $p_{k}$ is an immediate successor of transition $t_{j}$. When a Petri net is a model of some system, places correspond to its passive components, while transitions correspond to the active ones. In the case of models of biological systems transitions can model chemical reactions while places can be counterparts of their substrates and products, for example. Moreover, arcs correspond to causal relations between the passive and active components of the system. Each of the arcs is labeled by a positive integer number called a weight.

Places, transitions and arcs compose the structure of Petri nets but there are other important components bringing to the nets a dynamics, being one of their fundamental properties. These components are tokens residing in places and flowing from one place to another through transitions. This flow corresponds to a flow of information, substances, etc. in the modeled system. A vector of the numbers of tokens residing in every place is called marking of the net and corresponds to a state of the modeled system. The flow of tokens is governed by a simple rule called transition firing rule. According to it, a transition is enabled if the number of tokens residing in each of its pre-places is equal to or greater than the weight of an arc connecting such a place with the transition. An enabled transition can be fired what means that tokens can flow from its pre-places to its post-places. The number of flowing tokens is equal to the weight of an arc connecting the transition with the place. There are two exceptions to this rule, i.e., a transition without pre-places (the so-called input transition) is always enabled and a transition without post-places (the so-called output transition), when fired, does not produce any tokens. Transitions of these two types are usually used to model some connections of the system with its environment [14,15,16].

Petri nets have very intuitive graphical representation, which helps to better understand the structure and behavior of the net. In this representation places are shown as circles, transitions as rectangles or bars, arcs as arrows, and tokens as dots or positive integer numbers residing in places. If a weight of an arc is equal to 1, then it is usually not shown, otherwise, the number is associated with an arrow corresponding to a given arc.

#### 2.1.1. t-Invariants

Despite that the graphical representation is intuitive, it is not very well suited for the analysis of formal properties of the nets. For this purpose another representation, i.e., an incidence matrix, is used. In such a matrix $A={\left(a_{ij}\right)}_{n\times m}$, where *n* is the number of places and *m* is the number of transitions, rows correspond to places while columns correspond to transitions. Every entry $a_{ij}$ is an integer number equal to the difference between the numbers of tokens in place $p_{i}$ after and before firing transition $t_{j}$.

Petri nets have many interesting properties whose analysis can help to better understand the behavior of the net and the behavior of the modeled biological system. In our studies of the model we have put special attention to the analysis of the net invariants. There are two types of them, i.e., transition invariants (t-invariants) and place invariants (p-invariants). An invariant of the former type is vector $x\in N^{m}$ being a solution to the equation A·x=0. while an invariant of the latter type is vector $y\in N^{n}$, which is a solution of the equation

y·A=0.

With every t-invariant *x* there is associated its support, denoted by supp(x), being a set of transitions corresponding to positive entries in *x*, i.e., $supp\left(x\right)=\left\{t_{j}:x_{j}>0,j=1,2,\ldots ,m\right\}$. A t-invariant is minimal if its support does not properly contain a support of any other t-invariant, i.e., there is no another t-invariant $x^{\prime }$ such that $supp\left(x^{\prime }\right)\subset supp\left(x\right)$. A net is covered by t-invariants (i.e., CTI property holds, which is an important initial condition for an analysis based on t-invariants) if every transition belongs to a support of at least one t-invariant. A support of a p-invariant and a minimal p-invariant are defined analogously. Since every invariant can be expressed as a linear combination of the minimal ones usually only minimal invariants are analyzed.

If each transition $t_{j}$ belonging to a support of t-invariant *x* is fired a number of times equal to the invariant entry $x_{j}$ then the marking of the net will not be changed (i.e., the state of the system will remain unchanged). Moreover, the weighted number of tokens residing in places belonging to a support of p-invariant *y* is constant, where the weight for place $p_{i}$ is equal to the invariant entry $y_{i}$.

In the case of Petri nets being models of biological systems t-invariants correspond to some subprocesses occurring in the modeled system; t-invariants can be seen as biologically balanced processes. It means that firing of all transitions belonging to the t-invariant support a proper number of times will not change the state of the net (i.e., the tokens distribution in places). Obviously, these processes are composed of some elementary subprocesses modeled by individual transitions belonging to a support of a given t-invariant. Supports of various invariants can have non-empty intersections what means that the subprocesses modeled by these invariants are composed of some common elementary processes. Hence, the subprocesses can interact with each other. Therefore, the analysis of similarities between t-invariants may lead to the discoveries of some new interactions or dependencies between the subprocesses of the modeled system.

#### 2.1.2. MCT Sets

As we have explained before, a support of a t-invariant is a set of transitions. Such a set defines some minimal subnet representing a subprocess in the biological system. Within the supports some common sets of transitions can be identified and they are called Maximal Common Transition (MCT) sets. Such an approach is especially valuable when the number of t-invariants is too high to analyze them individually (this is the case for the presented model, with 2588 minimal t-invariants). MCT sets contain transitions being elements of supports of exactly the same t-invariants. Each MCT set corresponds to some functional module of the modeled system whose biological meaning should be determined. It is easy to see that a support of every t-invariant is a collection of some MCT sets (some of them may contain only one element and are called *trivial MCT sets*). Hence, the results of the t-invariant clustering can be analyzed in the context of dependencies among MCT sets [17].

#### 2.1.3. t-Clusters

In order to find such similarities, t-invariants can be grouped into structures called t-clusters (consisting of similar t-invariants and interpreted as biological functional modules) [17]. As a general rule for clustering one can assume that the elements of a given t-cluster (i.e., a set of transitions) are more similar to each other than to elements of any other t-cluster. The similarities between t-invariants are looked for among elements of each t-cluster [18,19,20,21]. Such clusters can be calculated using standard clustering methods (c.f. [20]). However, some important decisions when calculating clusters must be made. The first two of them concern the selection of clustering (joining) algorithm and a similarity measure. A proper similarity measure must take into account that in a case of t-invariants, their non-zero entries correspond to distinct, different transitions in the net. The third decision concerns the selection of the number of clusters that must divide t-invariants (representing system processes) into biologically relevant groups. To evaluate the clusters we have used the Mean Split Silhouette (MSS) evaluation index [22] to identify the best clustering with the optimal number of t-clusters (c.f. [20]). After comparing multiple sets of clusters being the results of combinations of such aforementioned decisions, we have decided that our analysis will rely on the clusters obtained using the Unweighted Pair Group Method with Arithmetic Mean (UPGMA) joining algorithm and the correlated Pearson similarity measure, which seems to usually give best results in t-clusters analysis of Petri net [17].

#### 2.1.4. Knockout Analysis

Knockout analysis has been performed in two different ways. The first one is based on t-invariants as described in [23], the second on the net simulation. For easier distinction between them the first one will be called *invariants knockout* or simply *knockout*, while the second one will be called *simulation knockout*.

In the invariants knockout, some selected transitions can be excluded from the model and the remaining invariants examined (i.e., the ones without the chosen, knocked out transitions in their supports). The remaining invariants may not fully cover the net. Therefore transitions that are not covered due to the knockout of some other ones are considered disabled. They are classified as dependent on the ones that have been initially knocked out. From the biological point of view it is interesting to detect which parts of the model will be affected by the knockout of the selected transitions. It can also be investigated which transitions should be knocked-out to achieve a desired model behavior.

The second type of knockout involves the simulation of the net and analyzing tokens distribution in places when some transitions have been knocked out in the model. In this type of approach a series of simulations are being performed, all starting from the same initial marking and ending after achieving the same number of steps. During a normal net simulation (i.e., without anything knocked out) an enabled transition has 50% chances for being fired. Additionally, multiple transitions can fire simultaneously in the same step if the number of tokens in all their pre-places allows it. Fired transitions consume tokens from their pre-places and produce them in post-places in a number defined by the weights of proper arcs. The chances of firing for all of the transitions as well as the sum of all accumulated tokens in the net places are gathered and averaged, taking into account the number of simulations. A simulation knockout, on the other hand, is a type of simulation performed when some transitions are marked as knocked out. Such transitions will never fire, no matter how many tokens are present in their pre-places. Using this type of simulation an influence of a knockout of some important reactions on the rest of the model can be studied.

**Figure 2:**
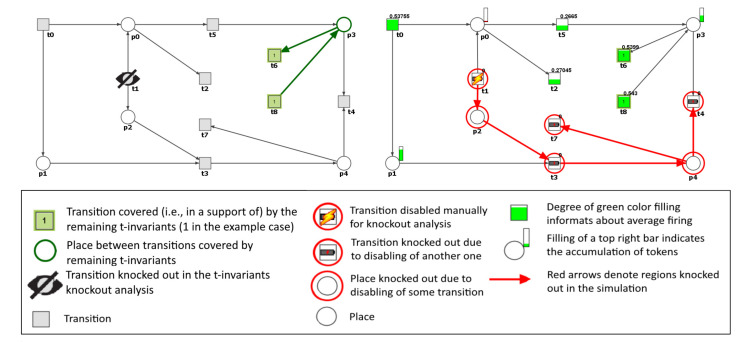
Example results of t-invariants knockout (left) and simulation knockout (right). In the left picture $t_{0}$ and $t_{3}$ (in blue) belong to the same MCT as $t_{1}$, while transitions covered in black are knocked out. Only $t_{6}$ and $t_{8}$ remained active, i.e., they are in the support of unaffected t-invariant. In the right picture the area colored in red do not function due to lack of tokens in $p_{2}$ caused by $t_{1}$ knockout. Other transitions still work and their average firing is given as a value above them. For working places the filling of a small bar represent total accumulation of their tokens in the simulation.

As an example a simple net is given in Figure 2.

In the left part of Figure 2 there are results from the first type of knockout approach, proposed in [23]. The net is covered by 6 t-invariants: $x_{0}=\left\{0,0,0,0,0,0,1,0,1\right\}$, $supp\left(x_{0}\right)=\left\{t_{6},t_{8}\right\}$, $x_{1}=\left\{1,1,2,1,0,0,0,1,0\right\}$, $supp\left(x_{1}\right)=\left\{t_{0},t_{1},t_{2},t_{3},t_{7}\right\}$, $x_{2}=\left\{1,1,0,1,0,2,2,1,0\right\}$, $supp\left(x_{2}\right)=\left\{t_{0},t_{1},t_{3},t_{5},t_{6},t_{7}\right\}$, $x_{3}=\left\{1,1,2,1,1,0,0,0,1\right\}$, $supp\left(x_{3}\right)=\left\{t_{0},t_{1},t_{2},t_{3},t_{4},t_{8}\right\}$, $x_{4}=\left\{1,1,1,1,1,1,0,0,0\right\}$, $supp\left(x_{4}\right)=\{t_{0}$, $t_{1}$, $t_{2}$, $t_{3}$, $t_{4}$, $t_{5}\}$, $x_{5}=\left\{1,1,0,1,1,2,1,0,0\right\}$, $supp\left(x_{5}\right)=\left\{t_{0},t_{1},t_{3},t_{4},t_{5},t_{6}\right\}$. Of these, five ($x_{1}$–$x_{5}$) contain transition $t_{1}$ in their supports and its knockout will disable these invariants. In other words, without the $t_{1}$ such processes represented by t-invariants will no longer be balanced, e.g., without $t_{1}$, transition $t_{3}$ will in theory consume tokens from $p_{2}$ (if any will be available) which will never be replaced without $t_{1}$ being active. Transitions $t_{6}$ and $t_{8}$ belong to the support of t-invariant $x_{0}$, which does not contain $t_{1}$ in its support and as a result they are not affected by the $t_{1}$ knockout. The process represented by $x_{0}$ is the only one that is still balanced in a scenario when $t_{1}$ does not function.

The right part of the Figure 2 shows an example of a simulation knockout. One can see that knockout of $t_{1}$ completely stops tokens production in $p_{2}$, and as a result $t_{3}$, $t_{4}$ and $t_{7}$ will never be enabled in such a scenario. The other transitions can still fire and their average chance of firing gathered in a simulation is given as a value above their symbol. Average tokens residing in each place in comparison to other places is given as a partly filled bar slightly above and to the right of each place symbol.

### 2.2. Description of Essential Hypertension-Related Phenomena That Were Taken into Account for Building the Model

This subsection shows the modeled disorder, divided into thematic blocks with their detailed features, along with the corresponding place and transition symbols that are included in the model. Both places and transitions are determined by the notation $p_{a}$ and $t_{b}$, where *a* and *b* are their respective numbers from Table 1 and Table 2.

**Figure 3:**
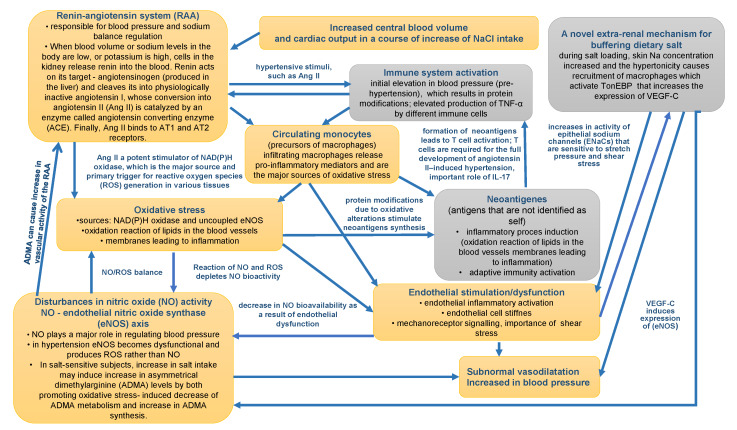
The diagram of the role of inflammation and immunity in essential hypertension. The orange blocks represent known factors affecting primary hypertension. Gray blocks include processes/factors whose involvement in hypertension is increasingly recognized.

In the proposed model there was no single overarching process. It was built based on several main processes that were introduced to the model based on current medical knowledge, clinical experience of the authors and some research hypotheses. To make the model easier to understand Figure 3, which shows a block diagram of factors/processes underlying essential hypertension and their interrelationships in the model, is presented.

#### 2.2.1. RAA System Pathway, in the Liver, Lungs and Kidney

RAA ($t_{0}$, $t_{1}$, $t_{2}$, $t_{5}$, $t_{10}$) is a physiological system that regulates blood pressure and fluid balance in the body [36]. First, cells in the kidney release the enzyme, prostaglandin (PGE2 ($p_{10}$)) and sympathetic stimulation under stress ($t_{63}$). Renin catalyzes the conversion of angiotensinogen ($p_{0}$) to angiotensin I ($p_{1}$). Then angiotensin-converting enzyme (ACE in lungs ($p_{3}$)) converts angiotensin I ($p_{1}$) into renin ($p_{9}$). This process is stimulated by decreased blood flow to the kidneys ($t_{10}$), prostacyclin (PGI2 ($p_{10}$)) and angiotensin II ($p_{2}$). Angiotensin II acts via at least four different subtypes of angiotensin receptors (ATRs). In the presented model we included two of them, whose roles are fairly well-documented, i.e., type 1 (AT1R ($p_{6}$)) and type 2 (AT2R ($p_{42}$)). High level of angiotensin II ($p_{2}$) through its interactions with the AT1R together with tumor necrosis factor alpha (TNF alpha ($p_{39}$, $p_{38}$, $p_{5}$)) and endothelin 1 (ET1 ($p_{7}$)) participates in the activation of NADPH oxidase ($t_{3}$, $t_{17}$, $p_{18}$) resulting in increased generation of superoxide (reactive oxygen species (ROS) ($t_{3}$, $p_{18}$, $t_{17}$, $p_{16}$, $t_{67}$)). Next, from the biradical reaction of superoxide ($p_{16}$) and NO ($p_{19}$) peroxynitrite ($p_{51}$) is formed. This reaction depletes NO bioactivity ($p_{51}$, $t_{71}$, $p_{22}$, $t_{73}$ and $p_{19}$) and promotes hypertension [24]. Opposing to the vasoconstrictor action of AT1R ($p_{6}$), angiotensin II ($p_{2}$) mediates vasodilation through the AT2R pathway. There is a link between AT2R ($p_{42}$) and vasodilators including NO ($p_{49}$) and bradykinin ($p_{29}$) [29]. The kinin-kallikrein system creates bradykinin ($p_{29}$) by proteolytic cleavage of its high-molecular-weight kininogen (HMWK ($p_{34}$, $t_{33}$, $p_{33}$ and $p_{32}$)) precursor, whose synthesis is stimulated by chronic inflammation ($t_{35}$, $p_{35}$, $t_{34}$, $p_{33}$, $p_{34}$ and $p_{26}$). Bradykinin ($p_{29}$) mediates its actions via two different types of receptors, i.e., non-constitutively expressed bradykinin receptor type 1 (BR1 ($p_{28}$, $p_{27}$, $t_{29}$ and $t_{28}$)) and consitutively expressed bradykinin receptor type 2 (BR2 ($p_{4}$, $p_{30}$ and $t_{30}$)). BR1 can be induced by inflammation ($p_{28}$ and $t_{35}$) and it modulates cardiovascular functions of bradykinin during chronic local inflammation ($t_{36}$ and $t_{28}$). On the other hand, BR2 is constitutively expressed in many tissues, e.g., vascular endothelium and smooth muscle cells and it mediates most of the vascular functions of bradykinin ($t_{4}$) [27]. ACE also breaks down the vasodilator bradykinin ($p_{29}$) into inactive fragments.

#### 2.2.2. Activation of the Immune System and the Formation of Neoantigens, Occurring in the Vascular Endothelium

The immune system activation ($t_{11}$ and $t_{12}$) [34] is one potential mechanism by which inflammation ($t_{35}$) may promote hypertension. T-effector cells ($p_{12}$) interact with innate immune mechanisms ($p_{14}$) to increase the inflammatory response via production of many cytokines ($t_{36}$) and ROS ($p_{16}$). An activated lymphocytes, particularly T lymphocytes may play an ongoing chronic inflammation, which is a source of infective antigens or neoantigens—modified molecules that are no longer recognized as self ($p_{15}$, $t_{41}$) in the organism. This can occur in response to many disturbances, such as the release of a molecules that are generally intracellular, oxidative modification of proteins, lipids or nucleic acids, cleavage of proteins what exposes intramolecular sites normally not being available for immune attack [28] or influence of increased shear stress ($p_{40}$, $t_{43}$ and $t_{44}$). Neoantigens are processed in antigen presenting cells (APC ($p_{13}$, $p_{14}$ and $t_{13}$)) and presented within a major histocompatibility complex. They activate T cells that leave secondary lymphoid organs and are targeted to sites of inflammation, i.e., the kidney and vasculature. Lymphocytes migration ($p_{12}$, $p_{26}$) is stimulated, inter alia, by the chemokine regulated on activation, normal T-cell expressed and secreted (RANTES) ($p_{25}$), which production increases under the influence of hypertensive stimuli, like angiotensin II ($p_{2}$). Concomitantly, in hypertension, an increased expression of CCR5 (receptor for RANTES ($t_{26}$ and $p_{24}$)) is observed, what leads to the migration of lymphocytes into the perivascular space, where cytokines, such as tumor necrosis factor alpha (TNF alpha ($p_{5}$, $p_{38}$, $p_{39}$, $t_{68}$, $t_{38}$, $t_{39}$ and $t_{27}$)), interferon gamma (IFN-gamma ($p_{37}$)) and interleukin 17 (IL-17 ($t_{50}$, $p_{36}$)) are released [37,38]. T cell derived signals like IL-17 promote an entry of other inflammatory cells, such as macrophages. These inflammatory cells release cytokines that cause vasoconstriction and promote sodium and water absorption, ultimately leading to severe hypertension [11].

#### 2.2.3. Novel Extra-Renal Mechanism for Buffering Dietary Salt in the Interstitium of the Skin—Probably an Additional System Affecting the Response to Salt Load and Blood Pressure in Humans

Recent discoveries have shown that macrophages act as local sensors and regulators of electrolyte composition in the interstitium of the skin [39]. The electrolytes in the skin do not readily equilibrate with plasma, and hence escape homeostatic control in the kidneys. Titze and colleagues [35,37] found that the traditional concept of the sodium homeostasis regulation, determining the level of the body’s volumes and blood pressure in the human organism, is much more complicated than it has been expected. Sodium can be stored without accumulation of water in the subdermal interstitium at hypertonic concentration via interactions with proteoglycans ($p_{41}$). This becomes a stimulus for mononuclear phagocyte system (MPS) ($p_{43}$) for the inflow to the interstitial space, under a local hypertonia (induced by sodium concentration), and for secreting TonEBP ($t_{57}$) that activates VEGF-C ($t_{58}$, $p_{31}$). VEGF-C can bind to one of its two canonical receptors. If it binds to VEGFR3 ($p_{45}$), then it leads to hyperplasia of lymph capillaries ($t_{60}$). If it is bound to VEGFR2 ($p_{31}$, $t_{62}$), it enhances production of NO. In addition, there is non-canonical signaling, i.e., VEGF-independent activation of VEGF receptors, in a course of excessive production of ROS ($p_{16}$ and $t_{69}$) [12,32].

#### 2.2.4. NO-eNOS Axis Affecting the Vascular Endothelium

NO ($p_{40}$) is released from endothelial cells and plays an important role in preserving the endothelial vasodilatation and inhibiting the vasoconstriction triggered by angiotensin II ($t_{65}$). It is recognized as one of the major mediators of the maintenance of vascular homeostasis. A decrease in bioavailability of NO ($t_{46}$ and $t_{73}$) is associated with endothelial dysfunction, which can alter the rates of synthesized and degraded vasoconstrictors and vasodilators leading to hypertension. NO is derived from endothelial nitric oxide synthase (eNOS) in the vessels in the presence of the cofactor tetrahydrobiopterin (BH4 ($t_{71}$)) and mediated through a cGMP-dependent downstream signaling cascade. In general, increased eNOS expression is considered to be beneficial. However, in various pathophysiological conditions, the function of eNOS is altered and it produces superoxide instead of NO, which is associated with reduced endothelium-dependent vasodilatation. This state is referred to as the uncoupled state of eNOS ($t_{24}$, $p_{22}$) [31] and is aggravated by asymmetric dimethylarginine (ADMA ($t_{21}$, $p_{21}$)), AT1R ($p_{6}$) and high levels of angiotensin II ($p_{2}$). Peroxynitrite ($p_{51}$, $t_{70}$) also uncouples eNOS by oxidation of BH4 which serves as a critical cofactor for eNOS ($p_{51}$, $t_{71}$ and $p_{22}$) [30]. Consequently, the loss of NO bioactivity associated with increased vascular superoxide plays a potentially important role in the pathogenesis of hypertension ($t_{49}$, $p_{17}$ and $t_{48}$).

## 3. Results and Discussion

### 3.1. The Model Presentation and the Results of Its Formal Analysis

**Figure 4:**
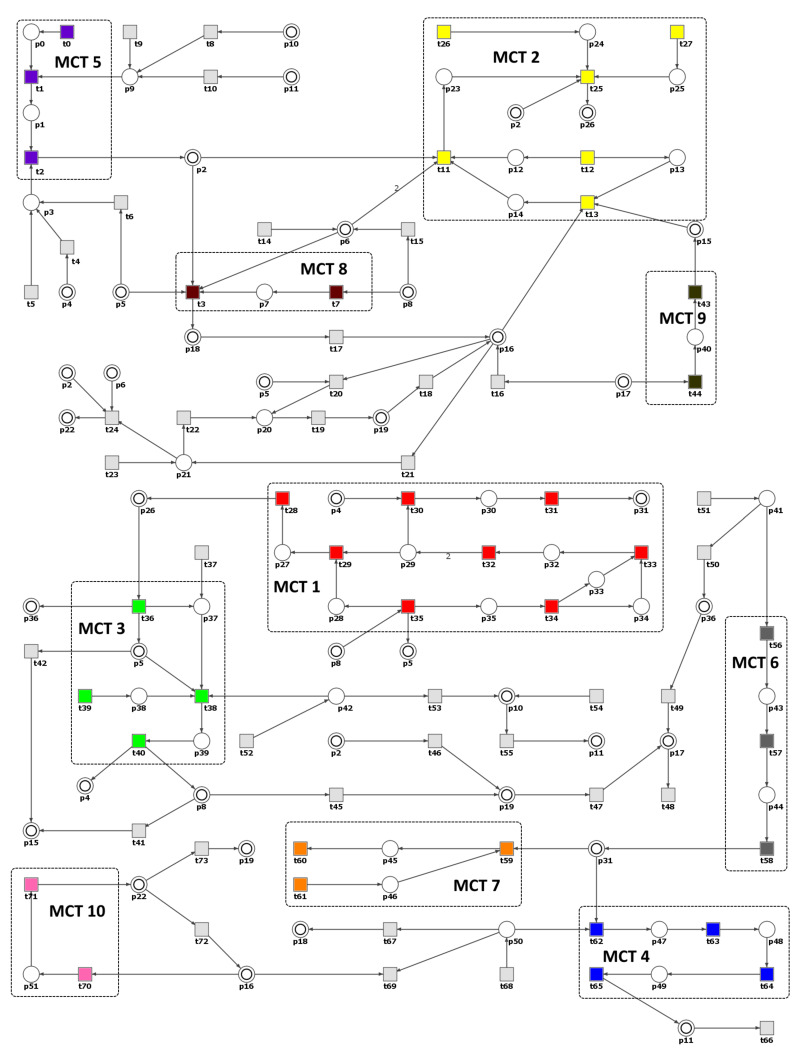
The Petri net based model. Non-trivial Maximal Common Transition (MCT) sets are marked by accordingly labeled rectangles. The transitions within a given MCT sets are shown with different colors. Logical places are shown as two concentric circles and are denoted by their names. The places and transitions are represented by both their names and numbers.

The proposed Petri net based model is in Figure 4 and is available at http:/www.cs.put.poznan.pl/arybarczyk/Hypertension/.

It contains 52 places and 74 transitions whose names are listed in Table 1 and Table 2, respectively. In the model, some places shown as two concentric circles exist in multiple copies, which are logically identical (e.g., $p_{13}$). Those places are called logical ones and their role is to improve model readability and simplify the visualization of connections between vertices. The net does not contain any information connected with the reaction speed nor exact amounts of reactants and products. However, important and interesting information about the described biological process can be derived from the structural analysis of the net [14,40]. Hence, the model itself is a very significant result.

The studied net is pure, i.e., there are no two nodes connected in both directions. The model is also not ordinary (i.e., there are weights greater than 1), because not every stoichiometric coefficient of each modeled reaction is equal to 1. Furthermore, the net is connected but not in a strong sense. It means that there exists an undirected path between any two places, but there may not be a directed path between them, what implies that there are no independent processes within the analyzed model. Moreover, the net is not structurally conflict-free because it contains places with two or more outgoing arcs. This entails that there are reactions sharing at least one substrate. Since the net is not ordinary, also the property of non-blocking multiplicity does not hold. The model contains neither input places nor output places (i.e., places without pre-transitions or post-transitions, respectively), but there exist a few input and output transitions. The net is also unbounded since there are no upper bound on the number of tokens.

The model contains 2588 minimal t-invariants covering all transitions (i.e., each transition belongs to a support of at least one t-invariant) and no p-invariant. The smallest supports of t-invariants consist of three transitions, e.g., a support of t-invariant $x_{2}$ includes transitions $t_{70}$, $t_{71}$ and $t_{72}$. The five largest supports of t-invariants, $x_{1477}$, $x_{1709}$, $x_{1728}$, $x_{1773}$ and $x_{2511}$, contain 44 transitions each. Transitions $t_{0}$ (angiotensinogen synthesis), $t_{1}$ (angiotensinogen with renin binding) and $t_{2}$ (angiotensinogen I with ACE binding) are of crucial importance for the network behavior because they occur in more than 98% of all supports of t-invariants. Except for five t-invariants, supports of all other ones contain input transitions and apart from 131 t-invariants, supports of all other contain output transitions.

In the net there are 10 non-trivial MCT sets listed in Table 3. All of them represent connected subnets and give a better insight into understanding the analyzed phenomenon. The results based on the clustering using UPGMA algorithm show that in the studied process one can distinguish one huge cluster $c_{4}$ covering almost the whole net (about 99% of t-invariants) and five clusters ($c_{1}$–$c_{3}$, $c_{5}$–$c_{6}$) containing small numbers of t-invariants. These clusters (six functional blocks), which are assigned a biological relevance, are presented in Table 4. This can be a unique feature of the analyzed biological process, where most of the subprocesses within the analyzed system are highly interconnected and interdependent. However, the results of the clustering are not satisfactory because it is very difficult to draw valuable biological conclusions without further analysis of the model.

As a next step of the analyses we conducted a knockout analysis. First, to analyze the knockout behavior of the model the impact of disabling some single activity (e.g., reaction) have to be determined. These activities are represented by MCT sets (non-trivial and the trivial ones, the latter being simply single transition). The impact of turning off a single activity on the analyzed net is measured as the percentage of t-invariants and what follows transitions affected by it. According to the authors of [23], the transitions in the model that are affected by a knockout of some other chosen transitions are those which belong only to the supports of the same t-invariants as the knocked-out ones (i.e., if a transition belongs to a support of at least one t-invariant which is not affected by a knockout of some other transitions, it is considered as being unaffected by it as well). We noticed, basing on the analysis supported by simulation knockout of the net, that in many cases the knockout of a given MCT set entails the inactivity of transitions that should not be affected as defined above. Therefore, we decided to conduct the knockout analysis based on both simulation knockout using Holmes software [41] and the approach described in [23] with the use of MonaLisa software [42]. All transitions detected by the simulation knockout to be inactive (along with the t-invariants they are involved in) and not included in the calculations of the selected activity knockout impact (according to MonaLisa) were included in the results presented in Table 5.

### 3.2. The Biological Questions We Answered on the Basis on the Knockout Analyzes

**Scenario** **1.** *The influence of RAA blockage on the essential hypertension development.*

The importance of the RAA in essential hypertension, with angiotensin II as a key player, is well established [43]. In the beginning, we imitated the action of angiotensin-converting enzyme inhibitors (ACEIs) or ATR blockers, drugs commonly used in the hypertension treatment.

**Figure 5:**
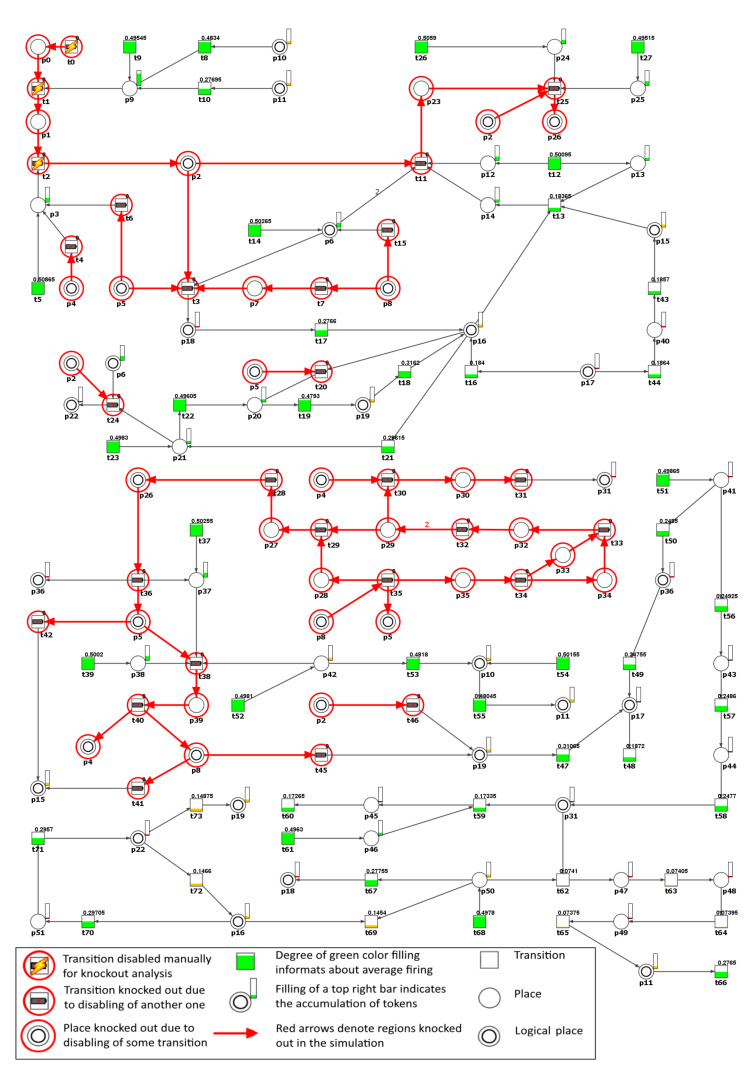
Graphical representation of the knockout results for the entire model, upon disabling of the transitions belonging to $m_{5}$. Inactive transitions, according to the simulation knockout, are marked with red circles. Active transitions are marked with rectangles filled with green or orange color which indicates whether the activity of a given transition has decreased (partially filled) or stayed intact (fully filled) as compared to the reference set. The results were obtained using Holmes software [41].

We found that according to Table 5, knockout of MCT set $m_{5}$—biological interpretation: angiotensinogen-angiotensin axis activation leading to angiotensin II formation, had a great impact on the net. To confirm this, we excluded from the model the transitions belonging to $m_{5}$ and simulated its behavior. Next, all of the transitions detected in the simulation knockout as inactive were also removed from the network and the remaining t-invariants were further investigated. Figure 5 shows the simulation knockout impact of the transitions belonging to $m_{5}$, related to the emergence of an essential hypertension activation through angiotensinogen-angiotensin axis.

The result of the analysis of the $m_{5}$ knockout impact on the other transitions in the model is presented in Table 6 whereas on the places in Table 7. It can be easily noticed that $m_{5}$ knockout indirectly influenced place $p_{8}$ (C-reactive protein (CRP), see Table 7) through the inactivation of transition $t_{40}$ (acute phase reaction, see Table 6 and Figure 5).

This relationship between RAA blockade and the lack of CRP and TNF alpha (place $p_{5}$, see Table 7) in the net, shows that the efficacy of ACEIs used in cardiovascular diseases was not only in the inhibition of angiotensin II, but partly in the anti-inflammatory properties of these drugs.

Similar results were also reported by other researchers [44,45]. Some of them observed a decrease in the concentration of pro-inflammatory markers, such as TNF alpha, or CRP in patients receiving ACEIs, even their blood pressure did not decrease during this therapy [46,47]. Accurate evaluation of the effect of RAA on the inflammatory process, especially in the course of cardiovascular diseases, requires further study. Our study indicates the validity of such research.

Moreover, our results reveal that blocking RAA showed a much deeper effect than just (1) blocking the formation of angiotensin II and (2) anti-inflammatory properties. This blockade affected transitions contained in four of MCT sets, i.e.,

$m_{1}$ (the initiation of blood coagulation and bradykinin production by the kallikrein-kinin system),

$m_{2}$ (lymphocytes T activation in hypertension as a part of immune system defense),

$m_{3}$ (the participation TNF alpha in the activation of the acute phase response in the course of hypertension) and,

$m_{8}$ (activation of a key enzymes of an oxidative stress (NADPH oxidases) through AT1 receptor) (see the Table 8).

This led to the (3) blockade of the initiation of blood coagulation (knockout of $m_{5}$ inactivates, among others, transitions belonging to $m_{1}$, like $t_{34}$ (increase in HMWK with zinc and XII factor) and inhibition of the places, like $p_{34}$ (HMWK with zinc, where HMWK is a circulating plasma protein which participates in the initiation of blood coagulation and the generation of the vasodilator bradykinin via the kallikrein-kinin system), $p_{33}$ (XII factor—the starting point of the intrinsic pathway in coagulation cascade), see the Table 7. Based on these results, it can be concluded that ACEI, blocking the initiation of coagulation, may play an essential role in reducing the risk of atherothrombotic events, as observed in some studies [48,49]. Our results can help explain the possible mechanisms by which ACEIs have such properties.

In addition, our results revealed that, by blocking angiotensin II action, activation of lymphocytes was affected (knockout of $m_{5}$ inactivates, among others, transitions belonging to $m_{2}$, like $t_{11}$ (T lymphocytes activation and proliferation via AT1R), $t_{12}$ (the immune system activation via AT1R), $t_{13}$ (APC with neoantigens binding), $t_{25}$ (migration of Th lymphocytes into the blood vessels), $t_{26}$ (increased expression of CCR5) and $t_{27}$ (RANTES influenced by TNF axis)). In turn, we observed that blocking RAA, has an impact on the TNF alpha pathway, (knockout of $m_{5}$ inactivates, among others, transitions belonging to $m_{3}$ like $t_{36}$ (the local release of cytokines—local inflammation), $t_{38}$ (TNF alpha with TNFR1 binding), $t_{39}$ (TNFR1 expression) and $t_{40}$ (acute phase reaction)). If we remember that elevated angiotensin II and other factors, such as oxidative stress, promote the formation of TNF alpha, and hypertension is considered to be a low-grade inflammation characterized by the presence of various proinflammatory cytokines, it is not surprising that the RAA blocking properties we indicate, showed how important drugs block the action of angiotensin II. To get a better insight into the axis: TNF alpha, renal function and blood pressure, see [50]. Moreover, we found, as other researchers [44,51,52,53], that angiotensin II is a potent prooxidant because it induces the production of superoxide anions and activates the prooxidant NADH/NADPH signaling pathways. The knockout of $m_{5}$ in our model, inactivated, among others, transitions $t_{3}$ (NADPH oxidase activation via AT1R) and $t_{7}$ (synthesis and stimulation of ET1 release) belonging to $m_{8}$ (activation of key enzymes of an oxidative stress (NADPH oxidase) through AT1 receptor) (see Table 8).

Finally, we revealed that, according to the results from Table 7, by blocking angiotensin II action, endothelin 1 (ET1, $p_{7}$), a potent vasoconstrictor, proinflammatory and proliferative endothelial cell-derived peptide that is of significant importance in the regulation of vascular function, was inhibited.

Our results confirmed that the treatment of essential hypertension with RAA inhibitors seems to be a good treatment strategy. The knockout of transition $t_{5}$ (ACE synthesis by vascular endothelium) which simulates the action of ACEIs, being the drugs that seem to provide clinical efficacy for the treatment of hypertension, is the most important in the model, because it causes knockout of 99.73% of all of the t-invariants in the modeled process (see Table 5). Next, regarding the importance of the processes, which we blockade, is $m_{5}$ (angiotensinogen-angiotensin axis activation leading to angiotensin II formation), and in the third place is $t_{14}$ (AT1R source). All of the mentioned processes refer to RAA inhibition. However, we should remember that in some hypertensive patients, this treatment may be insufficient (see Figure 5) because of the existence of other alternative pathways, what leads to maintaining essential hypertension.

Considering the potential benefits of RAA blocking, it seems that a commonly used antihypertensive therapy that inhibits the formation or action of angiotensin II, although very important, may not be sufficient in patients with essential hypertension necessary to control blood pressure completely. It is shown, based on our model analysis, that despite the RAA blocking, the essential hypertension is still present (see Figure 5). The exact explanation of this coincides with the answer to the next question.

*Is the local inflammatory process the only factor that can lead to essential hypertension?*

In our model we assumed that an inflammation, apart from activation of the RAA system and enhanced local production of angiotensin II, is a key feature that contributes to the initiation and progression of the cardiovascular disorders, including essential hypertension. This concept is fully supported by recent studies. However, it is not obvious whether the inflammation itself can promote hypertension. On the other hand, there are some studies on immunosuppressive drugs that could potentially be used to treat hypertension in patients with multidrug-resistant hypertension (immunosuppression attenuates hypertension in rats) [26,28].

The further analysis of $m_{5}$ knockout shows that the following transitions in the net became inactive according to the simulation knockout (see Table 6): $t_{11}$ (T lymphocytes activation and proliferation via AT1R), $t_{25}$ (Th lymphocytes migration into the blood vessels) and $t_{36}$ (the local release of cytokines (local inflammation)). These transitions are the crucial ones in the model since they are associated with the inflammation process leading to hypertension.

As one can see in Figure 5, transitions $t_{36}$ and $t_{50}$ (induction of Th17) directly corresponded to pro-inflammatory cytokine IL-17 (place $p_{36}$). Transition $t_{36}$ (which belongs to supports of 2505 of 2588 t-invariants, what reveals an important role of local inflammation in primary hypertension) become inactive, while transition $t_{50}$ (belonging to supports of eight t-invariants) is still active (because it belongs to support of two t-invariants remaining after $m_{5}$ knockout) and its activity stays almost unchanged (see Table 6). Summarizing this part of the analysis, it can be seen that blocking the RAA ($m_{5}$) does not affect the induction of Th 17 ($t_{50}$) and, what comes with it, the activated Th 17 cells still produce IL-17 cytokine, which is critical in the hypertensive process [38]. It promotes ROS production in the vascular smooth muscles and kidney, leading to vasoconstriction, sodium retention, and ultimately, severe hypertension (these factors determine the contents of cluster $c_{5}$ in our study).

**Figure 6:**
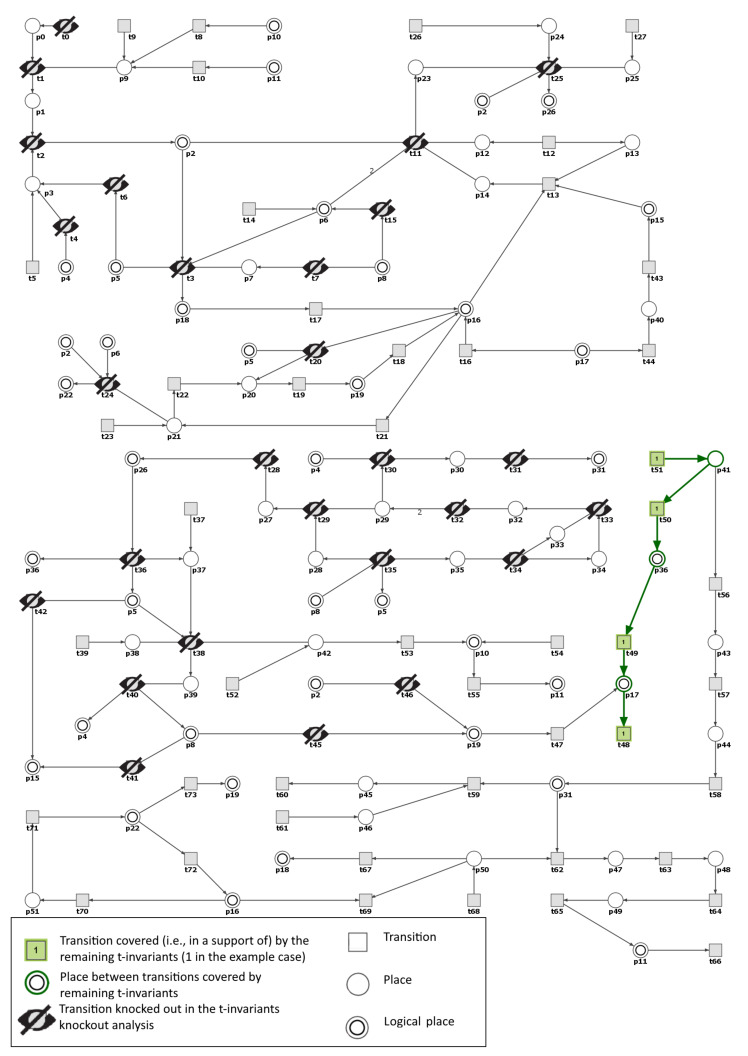
Graphical representation of the knockout impact of the transitions belonging to $m_{5}$—one of the two remaining t-invariants whose supports contain transition $t_{50}$ (a post-place of $t_{50}$ is $p_{36}$ corresponding to IL-17). Inactive transitions, according to the simulation knockout, are denoted with a crossed out black circles. Transitions belonging to the support of the t-invariant are marked with filled green rectangles. The results were obtained using Holmes software [41].

**Figure 7:**
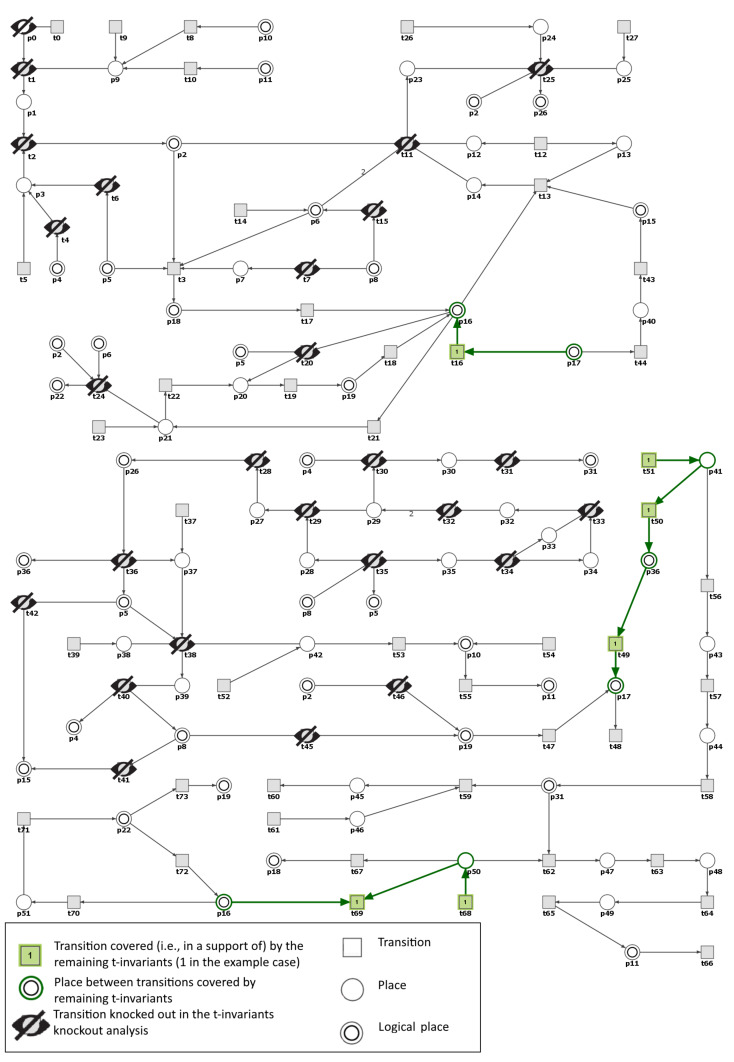
Graphical representation of the knockout impact of the transitions belonging to $m_{5}$—the second of the two remaining t-invariants whose supports contain transition $t_{50}$ (a post-place of $t_{50}$ is $p_{36}$ corresponding to IL-17). Inactive transitions, according to the simulation knockout, are denoted with a crossed out black circles. Transitions belonging to the support of the t-invariant are marked with filled green rectangles. The results were obtained using Holmes software [41].

Moreover, in the RAA knockout model the input transition $t_{51}$ (osmotically independent binding of Na in interstitium) is the only one, that is responsible for the $t_{50}$ activity in both t-invariants remaining active after $m_{5}$ knockout (see Figure 6 and Figure 7). These results confirm the importance of the discovery presented by Titze and colleagues [35,37] that sodium can be stored in the subdermal interstitium at hypertonic concentration through interactions with proteoglycans. In our model MCT set $m_{6}$ includes this path.

**Figure 8:**
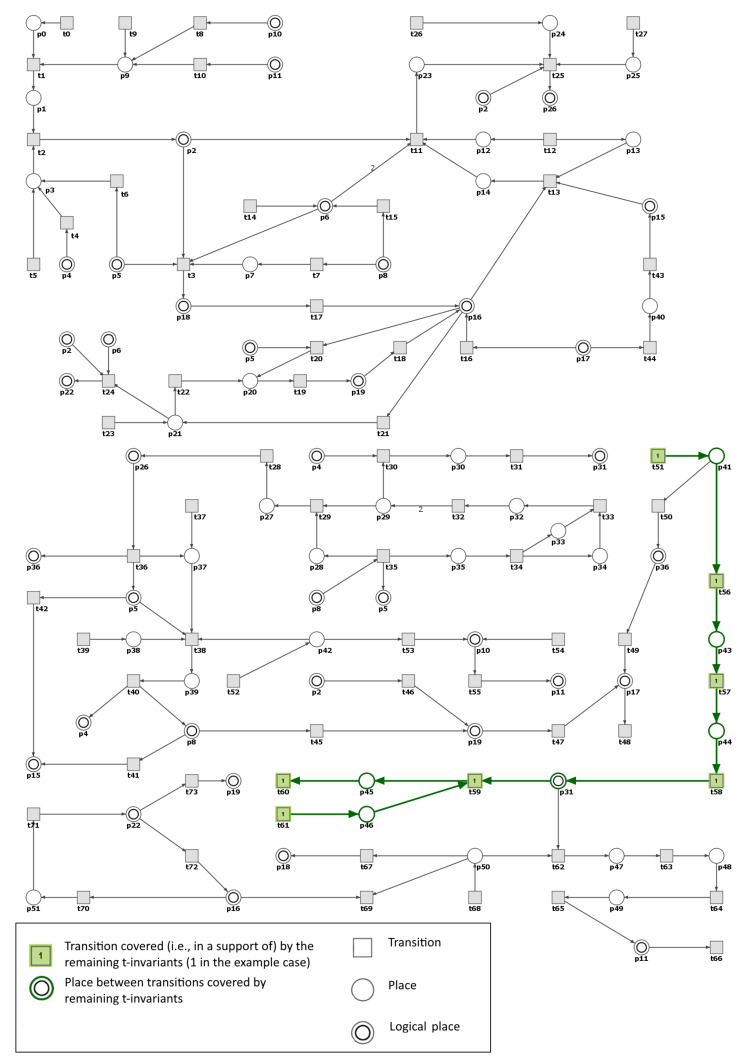
Graphical representation of the t-invariant corresponding to VEGF-C pathway. Transitions belonging to the support of the t-invariant are marked with filled green rectangles. The results were obtained using Holmes software [41].

If $t_{51}$ is also disabled in the RAA knockout model, then both $t_{36}$ and $t_{50}$ become inactive and what follows place $p_{36}$ (IL-17) is no longer carrying any tokens. It is worth noticing that $t_{51}$ belongs also to the support of t-invariant that corresponds to the process in which the hypertension is not developing (i.e., an invariant whose support contains transitions $t_{51},t_{56},t_{57},t_{58},t_{59},t_{60},t_{61}$, see Figure 8). The transitions belonging to the support of this t-invariant influence VEGF-C, place $p_{31}$). It is consistent with recent experimental findings showing that VEGF-C takes part in salt homeostasis and regulation of blood pressure. The VEGF-C-macrophage-lymphangiogenesis pathway is proved to play a role in the protection against developing hypertension in response to high sodium intake in animals [54]. This mechanism probably plays a very similar role in humans.

Thus, both RAA axis and maintenance of chronic inflammation are needed for the occurrence of hypertension in our model.

**Scenario** **2.** *Finding the processes that are responsible for homeostasis and regulation of blood pressure in humans.*

**Figure 9:**
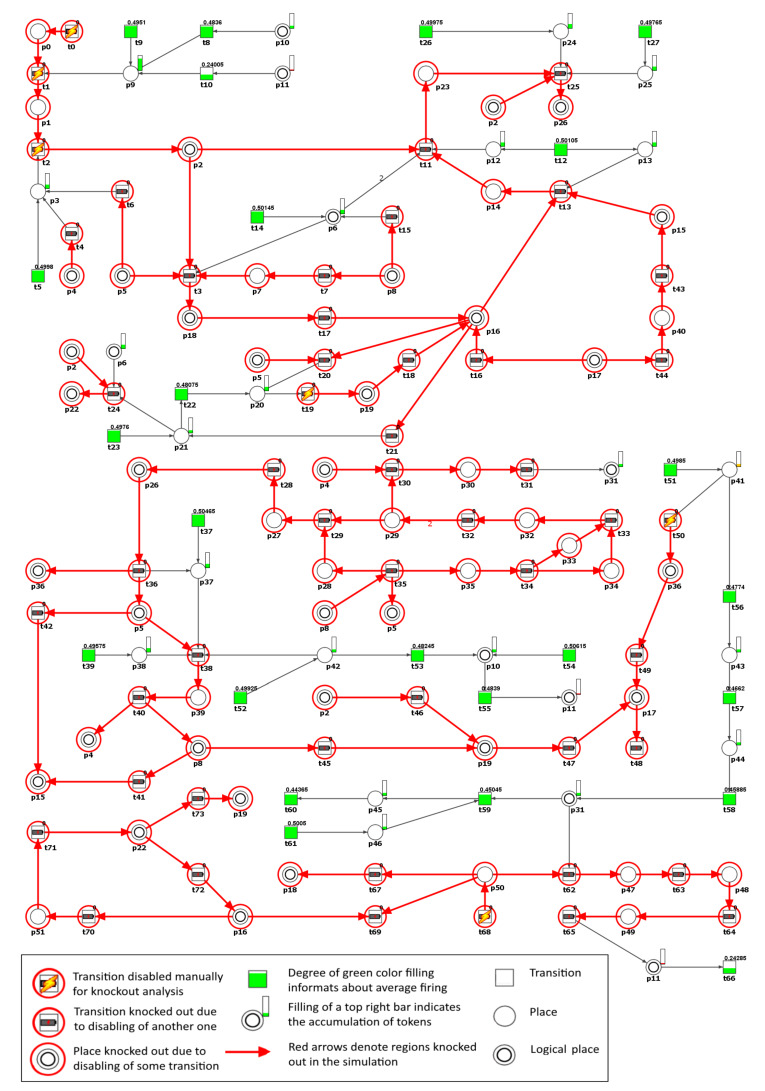
Graphical representation of the knockout results for the entire model, upon disabling of the transitions $t_{50}$, $t_{68}$, $t_{19}$ and those belonging to $m_{5}$. Inactive transitions, according to the simulation knockout, are marked with red circles. Active transitions are marked with rectangles filled with green or orange color which indicates whether the activity of a given transition has decreased (partially filled) or stayed intact (fully filled) as compared to the reference set. The results were obtained using Holmes software [41].

To answer this question we excluded from the model the following transitions: $t_{50}$ (induction of Th 17), $t_{68}$ (vascular endothelium VEGFR2 expression), $t_{19}$ (decreased NO synthesis by endothelial cells) and $m_{5}$ (angiotesinogen-angiotensin axis activation leading to angiotensin II formation). Figure 9 shows the knockout impact of these transitions. As a result three t-invariants remained and they correspond exactly to those clustered and described as $c_{1}$ and $c_{3}$ and presented in Table 4.

Our results showed that in silico knockout analyzes could be applied to examine and verify the dependencies and independencies of the processes characterized by MCT sets. In order to achieve it, transitions contained in a given MCT set are disabled and the model is simulated. Transitions that appeared to be inactive are further analyzed, whether they belong to any of the other non-trivial MCT sets. The whole process is repeated for each MCT set separately. These results are presented in Table 8.

They confirm that transitions grouped in some MCT sets, like $m_{2}$, $m_{3}$, $m_{5}$ and $m_{9}$, are the most crucial for the model, and their knockout excludes them alone and at least two other MCT sets (they represent the influence of the immune system, inflammatory process, RAA and neoantigens formation).

Summarizing, hypertension is a multifactorial process in which both the axis of the RAA, the inflammatory process with the induction of Th 17 and IL-17 synthesis and the influence of NO with its dual role (it dilates blood vessels, but also promotes oxidative stress) are fundamental.

## 4. Conclusions

Nowadays, we have at least two possibilities to study the phenomena underlying complex disorders. The first of them is based on the very precise analysis of a single phenomenon and its results can be easily verified by conducting the clinical and laboratory analyzes. This can simplify the building of the model and allow it to get more real precise data. However, such an approach also has its drawbacks because it allows one to analyze only a “slice of reality”. The second possibility of the studies is difficult to verify but allows us to assess the larger part of the studied phenomenon, so it is more real. Choosing it, one can detect dependencies that would be invisible if only a single path was examined. Unfortunately, this option also has its drawbacks because it is difficult to find numerical data from the available studies that would make the model more real, hence many rely on estimation. To the best of our knowledge, there is currently no study on essential hypertension in which many of the pathways underlying this disorder were analyzed at the same time. In our study, to simultaneously analyze more phenomena leading to hypertension, we applied a system approach based on the Petri net theory. First, we created a model, and then we blocked some of its subprocesses to draw conclusions. We obtained results, many of which were confirmed by other researchers in different studies. It means that our model was constructed properly because it allows us to obtain results in accordance with current knowledge. Our research enabled deep insight into hypertension and showed how many processes co-create hypertension and what their mutual relationships are. We found that at least several signaling pathways coexist together in the maintaining vascular homeostasis. The knockout of one of the studied sub-processes does not inhibit essential hypertension, what is consistent with clinical observations. It follows that RAA activation accompanied by the low-grade inflammatory process is necessary for the development of this type of hypertension.

It turned out that blocking RAA may affect: (1) formation of angiotensin II, (2) inflammatory properties (by influencing CRP), (3) initiation of blood coagulation, (4) generation of bradykinin, (5) activation of lymphocytes in hypertension, (6) participation of TNF alpha in the activation of the acute phase response, (7) activation of NADPH oxidase—a key enzyme of oxidative stress.

Concluding, we showed that despite the complex mechanisms underlying essential hypertension, we are able to reduce most of them with drugs blocking RAA activity. Interestingly, this blockade affects the studied disorder much more deeper than it would result from the role of angiotensin II itself, which blockade is crucial for the essential hypertension treatment. In this study we confirmed that there are links between RAA and other studied pathways showing how crucial is the role of inflammation and the immune system in maintaining hypertension. We observed that this mentioned treatment could affect both hypertension itself and coexisting phenomena such as inflammation, coagulation and oxidative stress, which in some patients may be essential for maintaining hypertension. It does not mean that all these disturbances must occur simultaneously, to the same extent in one patient, but shows that potentially all can have an effect.

We believe that our research may contribute to a better understanding of the pathology of hypertension. It may help to identify potential subprocesses, whose blocking will allow for better control of essential hypertension. The results presented in Table 5 revealed potential subprocesses together with their importance that may be blocked in the presented model of essential hypertension. The subprocesses, whose knockout impact is above 90%, seem to be the most promising, what is consistent with current knowledge. On the other hand, the blockade of the remaining transitions or MCT sets revealed these subprocesses, which could be rather the aim of the complementary therapies.

## Figures and Tables

**Table 1:** The list of places of the model.

Place	Biological Meaning	References	Place	Biological Meaning	References
$p_{0}$	angiotensinogen	[24,25,26]	$p_{26}$	T lymphocytes in adventitia and perivascular adipose tissue	[27,28]
$p_{1}$	angiotensin I	[24]	$p_{27}$	bradykinin and BR1 complex	[27]
$p_{2}$	high angiotensin II	[24,25,26,29,30]	$p_{28}$	BR1	[27]
$p_{3}$	ACE on vascular endothelium	[24,26]	$p_{29}$	bradykinin	[27,29]
$p_{4}$	BR2 in EC	[27]	$p_{30}$	bradykinin and B2R complex	[27]
$p_{5}$	TNF alpha	[24,25,26,28,31]	$p_{31}$	VEGF-C	[32]
$p_{6}$	AT1R	[24,25,26,30]	$p_{32}$	kalikrein	[27]
$p_{7}$	ET1	[24,25]	$p_{33}$	XII factor	[27]
$p_{8}$	C-reactive protein (CRP)	[25,26,33]	$p_{34}$	HMWK with zinc	[27]
$p_{9}$	prorenin-renin axis	[24,25]	$p_{35}$	damaged endothelium with platelets aggregation	[27]
$p_{10}$	high PGI2	[24]	$p_{36}$	IL-17	[11,28]
$p_{11}$	lower blood pressure	[24,27]	$p_{37}$	increase in IFN gamma	[28]
$p_{12}$	T lymphocytes	[11,26,34]	$p_{38}$	TNFR1	[24]
$p_{13}$	APC (macrophages, lymphocytes B and dendritic cells)	[11,28]	$p_{39}$	TNF alpha-TNFR1 complex	[24]
$p_{14}$	APC cells with neoantigens	[28,34]	$p_{40}$	shear stress	[28]
$p_{15}$	neoantigens	[28,34]	$p_{41}$	Na bound to glycosaminoglycans in interstitium	[35]
$p_{16}$	superoxide anion	[24,25,32,34]	$p_{42}$	AT2R	[24,29]
$p_{17}$	high blood pressure	[30]	$p_{43}$	attracted MPS	[35]
$p_{18}$	NADPH oxidase activated	[24,25]	$p_{44}$	TonEBP	[35]
$p_{19}$	low NO	[24,25,26]	$p_{45}$	VEGF-C and VEGFR3 complex	[32]
$p_{20}$	low eNOS coupled	[24,25,26]	$p_{46}$	VEGFR3	[32]
$p_{21}$	ADMA	[26,30]	$p_{47}$	VEGF-C and VEGFR2 complex	[32]
$p_{22}$	eNOS uncoupled	[24,26,30,31,33]	$p_{48}$	high eNOS coupled	[31]
$p_{23}$	Th lymphocytes	[34]	$p_{49}$	high NO	[29,31]
$p_{24}$	CCR5	[28]	$p_{50}$	VEGFR2	[32]
$p_{25}$	RANTES	[28]	$p_{51}$	peroxynitrite (ONOO anion)	[24,30]

**Table 2:** The list of transitions of the model.

Transition	Biological Meaning	References	Transition	Biological Meaning	References
$t_{0}$	angiotensinogen synthesis	[24,25]	$t_{37}$	IFN gamma synthesis as response for LPS	[26]
$t_{1}$	angiotensinogen with renin binding	[24,25]	$t_{38}$	TNF alpha with TNFR1 binding	[28]
$t_{2}$	angiotensin I with ACE binding	[24,25]	$t_{39}$	TNFR1 expression	[28]
$t_{3}$	NADPH oxidase activation via AT1R	[24,25]	$t_{40}$	acute phase reaction	[27,34]
$t_{4}$	ACE influenced by BR2	[27]	$t_{41}$	increase in neoantigens formation	[28]
$t_{5}$	ACE synthesis by vascular endothelium	[24,25]	$t_{42}$	neoantigens formation	[28]
$t_{6}$	ACE influenced by TNF alpha	[31]	$t_{43}$	neoantigens formation under shear stress	[28]
$t_{7}$	synthesis and stimulation of ET1 release	[24,25]	$t_{44}$	shear stress influenced by high blood pressure	[28]
$t_{8}$	stimulation of prorenin-renin axis caused by PGI2 and PGE2	[25]	$t_{45}$	reducing the activity of eNOS by CRP	[33]
$t_{9}$	sympathetic stimulation under stress	[24]	$t_{46}$	reduction of the bioavailability of NO	[24,31]
$t_{10}$	decrease in renal perfussion	[24,25]	$t_{47}$	vasoconstriction	[11]
$t_{11}$	T lymphocytes activation and proliferation via AT1R	[34]	$t_{48}$	hypertension	[30]
$t_{12}$	the immune system activation via inflammation	[34]	$t_{49}$	blood pressure increasing	[30]
$t_{13}$	APC with neoantigens binding	[28]	$t_{50}$	induction of Th 17	[28]
$t_{14}$	AT1R source	[24,25,26,30]	$t_{51}$	osmotically independent binding of Na in interstitium	[35]
$t_{15}$	increased AT1R at vascular smooth muscles	[24,25,26]	$t_{52}$	AT2R expression	[29]
$t_{16}$	superoxide anion generation via high blood pressure	[24,25]	$t_{53}$	AT2R mediated PGI2 production by endothelial cells	[29]
$t_{17}$	superoxide anion generation via NADPH oxidase	[24,25]	$t_{54}$	synthesis of PGI2 by endothelial cells	[24]
$t_{18}$	superoxide anion generation via low NO	[31]	$t_{55}$	lowering blood pressure by PGI2	[24]
$t_{19}$	decreased NO synthesis by endothelial cells	[31]	$t_{56}$	the immune system activation in interstitium under local hypertonic state	[35]
$t_{20}$	eNOS expression significantly attenuated by TNF alpha	[31]	$t_{57}$	TonEBP synthesis under local hypertonic state	[35]
$t_{21}$	induction of ADAMA synthesis by oxidative stress	[30]	$t_{58}$	VEGF-C activation	[32]
$t_{22}$	eNOS expression significantly attenuated by ADMA	[26]	$t_{59}$	VEGF-C with VEGFR3 binding	[32]
$t_{23}$	early hypertension or kidney disease	[26]	$t_{60}$	modification of lymph capillary network via VEGFR3	[32]
$t_{24}$	eNOS uncoupled formation	[30,31,33]	$t_{61}$	process in lymphatic endothelium	[32]
$t_{25}$	migration of Th lymphocytes into the blood vessels	[11]	$t_{62}$	VEGF-C with VEGFR2 binding (canonical signaling)	[32]
$t_{26}$	increased expression of CCR5	[28]	$t_{63}$	eNOS synthesis induction, calcium and phosphorylation dependent	[32]
$t_{27}$	RANTES influenced by TNF-TNFR axis	[28]	$t_{64}$	NO increase	[32]
$t_{28}$	ICAM1, VCAM1 and PECAM endothelial stimulation	[27]	$t_{65}$	muscles relaxation	[31]
$t_{29}$	bradykinin with BR1 binding	[27]	$t_{66}$	health	[32]
$t_{30}$	bradykinin with BR2R binding	[27]	$t_{67}$	NADPH oxidase activation via VEGFR2	[24]
$t_{31}$	stimulation of VEGF-C formation by bradykinin-B2R complex	[27,32]	$t_{68}$	vascular endothelium VEGFR2 expression	[32]
$t_{32}$	bradykinin formation	[27]	$t_{69}$	VEGF pathway (ligand independent, non canonical signaling)	[32]
$t_{33}$	prekalikrein and kalikrein formation	[27]	$t_{70}$	peroxynitrite (ONOO anion) formation	[24,30]
$t_{34}$	increase in HMWK with zinc and XII factor	[27]	$t_{71}$	oxidation of BH4 (eNOS cofactor)	[24,30,31]
$t_{35}$	chronic inflammatory process	[27,34]	$t_{72}$	superoxide anion synthesis by eNOS uncoupling	[33]
$t_{36}$	the local release of cytokines (local inflammation)	[27,34]	$t_{73}$	low NO synthesis	[24,31]

**Table 3:** The list of non-trivial MCT sets.

MCT-Set	Contained Transitions	Biological Interpretation
$m_{1}$	$t_{28}$, $t_{29}$, $t_{30}$, $t_{31}$, $t_{32}$, $t_{33}$, $t_{34}$, $t_{35}$	The initiation of blood coagulation, and the generation of bradykinin via the kallikrein-kinin system
$m_{2}$	$t_{11}$, $t_{12}$, $t_{13}$, $t_{25}$, $t_{26}$, $t_{27}$	Lymphocytes T activation in hypertension—as a part of immune system defense
$m_{3}$	$t_{36}$, $t_{38}$, $t_{39}$, $t_{40}$	The participation of TNF alpha in the activation of the acute phase response in the course of hypertension
$m_{4}$	$t_{62}$, $t_{63}$, $t_{64}$, $t_{65}$	Impact of VEGF-C—VEGFR2 axis on the nitric oxide synthesis and relaxation of vascular smooth muscles
$m_{5}$	$t_{0}$, $t_{1}$, $t_{2}$	Angiotensinogen-angiotensin axis activation leading to angiotensin II formation
$m_{6}$	$t_{56}$, $t_{57}$, $t_{58}$	Local activation of the immune system due to changes in the local hypertonia associated with the activation of VEGF-C
$m_{7}$	$t_{59}$, $t_{60}$, $t_{61}$	Impact of the VEGF-C—VEGFR3 axis on the lymphatic endothelium
$m_{8}$	$t_{3}$, $t_{7}$	Activation of key enzymes of oxidative stress (NADPH oxidases) through AT1R
$m_{9}$	$t_{43}$, $t_{44}$	The influence of shear stress on the formation of neoantigens and increased arterial blood pressure
$m_{10}$	$t_{70}$, $t_{71}$	Peroxynitrite formation as a part of oxidative stress signaling pathway

**Table 4:** The 2588 feasible t-invariants of the model clustered by Unweighted Pair Group Method with Arithmetic Mean (UPGMA) algorithm. In the two columns on the right side of the table, the processes contained in the clusters are listed. Processes are divided into non-trivial MCT sets and single transitions. The numbers presented in brackets are fractions (a number of supports of t-invariants from a given t-clusters containing a given process)/(a number of supports of t-invariants from all other t-clusters containing a given process)%. The lack of any number means that a given transition or MCT-set occurs only in supports of t-invariants being elements of a given t-cluster. The columns on the left side give the total number of t-invariants in the cluster, together with its biological interpretation.

Cluster No.	Biological Interpretation	No. of t-Invariants	Contained Processes	
			MCT-Sets	Single Transitions
$c_{1}$	The local changes in the interstitium due to fluctuations in the local independent osmotic sodium concentration	1	$m_{6}\left(0.9\right)$, $m_{7}\left(0.1\right)$	$t_{51}\left(0.86\right)$
$c_{2}$	Formation and effects of reactive oxygen species on hypertension	4	$m_{10}\left(13.3\right)$	$t_{16}\left(0.11\right)$, $t_{17}\left(0.13\right)$, $t_{18}\left(0.11\right)$, $t_{47}\left(0.20\right)$, $t_{48}\left(0.06\right)$, $t_{67}\left(0.39\right)$, $t_{68}\left(0.05\right)$, $t_{72}\left(0.24\right)$, $t_{73}\left(0.60\right)$
$c_{3}$	Processes mediated by PGI2, resulting in the reduction of blood pressure and maintenance of health state	2		$t_{52}\left(0.04\right)$, $t_{53}\left(0.13\right)$, $t_{54}\left(0.13\right)$, $t_{55}\left(0.33\right)$, $t_{66}\left(0.33\right)$
$c_{4}$	The influence of oxidative stress and inflammation on vascular endothelium in the course of arterial hypertension without changes regarding the lymphatic endothelium	2572	$m_{1}$, $m_{2}$, $m_{3}$, $m_{4}$, $m_{5}$, $m_{6}\left(99\right)$, $m_{7}\left(99.9\right)$, $m_{8}$, $m_{9}$, $m_{10}\left(87\right)$	$t_{4}$, $t_{5}$, $t_{6}$, $t_{8}$, $t_{9}$, $t_{10}$, $t_{14}$, $t_{15}$, $t_{16}\left(99.58\right)$, $t_{17}\left(99.60\right)$, $t_{18}\left(99.68\right)$, $t_{19}\left(98.47\right)$, $t_{20}$, $t_{21}\left(99.10\right)$, $t_{22}\left(95.89\right)$, $t_{23}\left(99.57\right)$, $t_{24}$, $t_{37}$, $t_{41}$, $t_{42}$, $t_{45}$, $t_{46}$, $t_{47}\left(99.41\right)$, $t_{48}\left(99.77\right)$, $t_{49}\left(99.92\right)$, $t_{50}\left(75.00\right)$, $t_{51}\left(97.41\right)$, $t_{52}\left(99.96\right)$, $t_{53}\left(99.87\right)$, $t_{54}\left(99.87\right)$, $t_{55}\left(99.67\right)$, $t_{66}\left(99.67\right)$, $t_{67}\left(98.84\right)$, $t_{68}\left(99.69\right)$, $t_{69}\left(99.50\right)$, $t_{72}\left(99.76\right)$, $t_{73}\left(99.40\right)$
$c_{5}$	Processes leading to hypertension, with particular emphasis on the role of IL-17	2		$t_{16}\left(0.11\right)$, $t_{48}\left(0.06\right)$, $t_{49}\left(0.08\right)$, $t_{50}\left(25.00\right)$, $t_{51}\left(1.72\right)$, $t_{68}\left(0.05\right)$, $t_{69}\left(0.12\right)$
$c_{6}$	Processes leading to hypertension, with particular emphasis on the effect of nitric oxide, the role of ADMA and VEGFR2	7		$t_{16}\left(0.21\right)$, $t_{17}\left(0.27\right)$, $t_{18}\left(0.21\right)$, $t_{19}\left(1.53\right)$, $t_{21}\left(0.90\right)$, $t_{22}\left(4.11\right)$, $t_{23}\left(0.43\right)$, $t_{47}\left(0.39\right)$, $t_{48}\left(0.11\right)$, $t_{67}\left(0.77\right)$, $t_{68}\left(0.21\right)$, $t_{69}\left(0.37\right)$

**Table 5:** The most important activities in the model according to their combinatorial knockout impact calculated based on both simulation knockout and the approach described in [23]. In the calculation of the knockout impact in the case of transitions, only the inactive ones, according to simulation knockout, were taken into account.

MCT-Set	Activity	Knockout Impact	Knockout Impact
$t_{5}$	ACE synthesis by vascular endothelium	33.78%	99.73%
$m_{5}$	angiotesinogen-angiotensin axis activation leading to angiotensin II formation	28.38%	99.34%
$t_{14}$	AT1R source	31.08%	99.23%
$m_{9}$	the influence of shear stress on the formation of neoantigens and increased arterial blood pressure	31.08%	99.23%
$m_{2}$	lymphocytes T activation in hypertension as a part of immune system defense	27.03%	98.92%
$t_{52}$	AT2R expression	22.97%	97.68%
$t_{49}$	blood pressure increasing	0.00%	96.87%
$m_{3}$	the participation TNF alpha in the activation of the acute phase response in the course of hypertension	22.97%	96.79%
$m_{1}$	the initiation of blood coagulation, and the generation of bradykinin via the kallikrein-kinin system	0.00%	92.39%
$t_{4}$	ACE influenced by BR2	0.00%	88.79%
$t_{68}$	vascular endothelium VEGFR2 expression	8.11%	74.03%
$t_{48}$	hypertension	0.00%	68.47%
$m_{4}$	impact of the VEGF-C—VEGFR2 axis on the nitric oxide synthesis and relaxation of vascular smooth muscles	0.00%	55.91%
$t_{37}$	IFN gamma synthesis as response for LPS	0.00%	53.40%
$t_{10}$	decrease in renal perfussion	0.00%	50.50%
$t_{15}$	increased AT1R at vascular smooth muscles	0.00%	43.51%
$t_{47}$	vasoconstriction	0.00%	39.26%
$m_{7}$	impact of the VEGF-C—VEGFR3 axis on the lymphatic endothelium	0.00%	38.68%
$t_{8}$	stimulation of prorenin-renin axis caused by PGI2 and PGE2	0.00%	38.25%
$t_{16}$	superoxide anion generation via high blood pressure	0.00%	36.51%
$t_{18}$	superoxide anion generation via low NO	0.00%	36.40%
$t_{24}$	eNOS uncoupled formation	0.00%	34.47%
$t_{45}$	reducing the activity of eNOS by CRP	0.00%	31.72%
$t_{69}$	VEGF pathway (ligand independent, non canonical signaling)	0.00%	31.11%
$t_{53}$	AT2R mediated PGI2 production by endothelial cells	0.00%	30.80%
$t_{54}$	synthesis of PGI2 by endothelial cells	0.00%	30.80%
$t_{17}$	superoxide anion generation via NADPH oxidase	0.00%	29.02%
$t_{23}$	early hypertension or kidney disease	0.00%	27.13%
$t_{6}$	ACE influenced by TNF alpha	0.00%	25.70%
$t_{66}$	health	0.00%	23.76%
$t_{55}$	lowering blood pressure by PGI2	0.00%	23.34%
$t_{42}$	neoantigens formation	0.00%	23.18%
$t_{73}$	increase in neoantigens formation	0.00%	19.36%
$t_{41}$	low NO synthesis	0.00%	19.32%
$t_{46}$	reduced bioavailability of NO	0.00%	19.24%
$m_{8}$	activation of a key enzymes of oxidative stress (NADPH oxidases) through AT1R	0.00%	19.13%
$t_{9}$	sympathetic stimulation under stress	0.00%	19.13%
$t_{72}$	superoxide anion synthesis by eNOS uncoupling	0.00%	16.27%
$t_{19}$	decreased NO synthesis by endothelial cells	0.00%	15.19%
$t_{21}$	induction of ADAMA synthesis by oxidative stress	0.00%	12.94%
$t_{67}$	NADPH oxidase activation via VEGFR2	0.00%	10.01%
$t_{20}$	eNOS expression significantly attenuated by TNF alpha	0.00%	9.81%
$t_{22}$	eNOS expression significantly attenuated by ADMA	0.00%	5.64%
$t_{51}$	osmotically independent binding of Na in interstitium	5.41%	4.48%
$m_{6}$	local activation of the immune system due to changes in the local hypertonia associated with an activation of VEGF-C	0.00%	4.21%
$m_{10}$	peroxynitrite formation as a part of an oxidative stress signaling pathway	0.00%	1.16%
$t_{50}$	induction of Th 17	0.00%	0.31%

**Table 6:** The impact of $m_{5}$ knockout. The columns on the right side of the table present each transition activity before and after the knockout of transitions belonging to $m_{5}$. The change in the activity in the case of each transition is shown in the last column. A positive value means that a given transition activity has increased while the negative one indicates that the transition activity has decreased. The transitions that were manually disabled are denoted as “(Offline)”. Those indicated by the simulation knockout as inactive due to $m_{5}$ knockout are marked as “(Knockout)”. Each entry in the table corresponds to the average value coming from 4000 simulation runs, each having 10,000 steps.

Transition	Biological Meaning	The Transition Activity in the Reference Set	The Transition Activity in the Set with $m_{5}$ Knocked out	Difference in the Transition Activity as Compared to the Reference and $m_{5}$ Knocked out Set
$t_{0}$	(Offline) angiotensinogen synthesis	50.00%	0.00%	−50.00%
$t_{1}$	(Offline) angiotensinogen with renin binding	49.44%	0.00%	−49.44%
$t_{2}$	(Offline) angiotensin I with ACE binding	49.05%	0.00%	−49.05%
$t_{3}$	(Knockout) NADPH oxidase activation via AT1R	0.60%	0.00%	−0.60%
$t_{4}$	(Knockout) ACE influenced by BR2	2.51%	0.00%	−2.51%
$t_{5}$	ACE synthesis by vascular endothelium	50.00%	50.00%	0.00%
$t_{6}$	(Knockout) ACE influenced by TNF alpha	3.50%	0.00%	−3.50%
$t_{7}$	(Knockout) synthesis and stimulation of ET1 release	0.63%	0.00%	−0.63%
$t_{8}$	stimulation of prorenin-renin axis caused by PGI2 and PGE2	48.32%	49.41%	1.09%
$t_{9}$	sympathetic stimulation under stress	49.98%	49.99%	0.01%
$t_{10}$	decrease in renal perfussion	27.82%	28.45%	0.63%
$t_{11}$	(Knockout) T lymphocytes activation and proliferation via AT1R	12.21%	0.00%	−12.21%
$t_{12}$	the immune system activation via inflammation	50.00%	50.01%	0.01%
$t_{13}$	APC with neoantigens binding	30.87%	19.04%	−11.83%
$t_{14}$	AT1R source	50.01%	50.01%	0.00%
$t_{15}$	(Knockout) increased AT1R at vascular smooth muscles	0.62%	0.00%	−0.62%
$t_{16}$	superoxide anion generation via high blood pressure	26.84%	19.06%	−7.79%
$t_{17}$	superoxide anion generation via NADPH oxidase	26.55%	27.67%	1.12%
$t_{17}$	superoxide anion generation via NADPH oxidase	26.55%	27.67%	1.12%
$t_{18}$	superoxide anion generation via low NO	43.17%	32.21%	−10.96%
$t_{19}$	decreased NO synthesis by EC	49.85%	49.42%	−0.43%
$t_{20}$	(Knockout) eNOS expression significantly attenuated by TNF alpha	2.31%	0.00%	−2.31%
$t_{21}$	induction of ADMA synthesis by oxidative stress	35.15%	30.04%	−5.11%
$t_{22}$	eNOS expression significantly attenuated by ADMA	49.97%	49.98%	0.02%
$t_{23}$	early hypertension or kidney disease	50.01%	49.99%	−0.02%
$t_{24}$	(Knockout) eNOS uncoupled formation	12.22%	0.00%	−12.22%
$t_{25}$	(Knockout) migration of Th lymphocytes into the blood vessels	11.80%	0.00%	−11.80%
$t_{26}$	increased expression of CCR5	50.00%	50.00%	0.00%
$t_{27}$	RANTES influenced by TNF-TNFR axis	50.00%	50.00%	0.01%
$t_{28}$	(Knockout) ICAM1, VCAM1 and PECAM endothelial stimulation	0.62%	0.00%	−0.62%
$t_{29}$	(Knockout) bradykinin with BR1 binding	0.62%	0.00%	−0.62%
$t_{30}$	(Knockout) bradykinin with BR2R binding	0.62%	0.00%	−0.62%
$t_{31}$	(Knockout) stimulation VEGF-C formation by bradykinin-B2R complex	0.62%	0.00%	−0.62%
$t_{32}$	(Knockout) bradykinin formation	0.62%	0.00%	−0.62%
$t_{33}$	(Knockout) prekalikrein and kalikrein formation	0.62%	0.00%	−0.62%
$t_{34}$	(Knockout) increase in HMWK with zinc and XII factor	0.62%	0.00%	−0.62%
$t_{35}$	(Knockout) chronic inflammatory process	0.62%	0.00%	−0.62%
$t_{36}$	(Knockout) the local release of cytokines (local inflammation)	12.42%	0.00%	−12.42%
$t_{37}$	IFN gamma synthesis as response for LPS	49.99%	50.01%	0.02%
$t_{38}$	(Knockout) TNF alpha with TNFR1 binding	3.13%	0.00%	−3.13%
$t_{39}$	TNFR1 expression	50.00%	49.99%	−0.01%
$t_{40}$	(Knockout) acute phase reaction in the liver	3.13%	0.00%	−3.13%
$t_{41}$	(Knockout) increase in neoantigens formation	0.63%	0.00%	−0.63%
$t_{42}$	(Knockout) neoantigens formation	3.50%	0.00%	−3.50%
$t_{43}$	neoantigens formation under shear stress	26.83%	19.06%	−7.78%
$t_{44}$	shear stress influenced by high blood pressure	26.84%	19.06%	−7.78%
$t_{45}$	(Knockout) reducing the activity of eNOS by CRP	0.62%	0.00%	−0.62%
$t_{46}$	(Knockout) reduced bioavailability of NO	12.22%	0.00%	−12.22%
$t_{47}$	vasoconstriction	43.15%	32.20%	−10.95%
$t_{48}$	hypertension	26.85%	19.06%	−7.79%
$t_{49}$	blood pressure increasing	37.40%	24.99%	−12.41%
$t_{50}$	induction of Th 17	25.00%	24.99%	0.00%
$t_{51}$	osmotically independent binding of Na in interstitium	50.00%	50.00%	0.00%
$t_{52}$	AT2R expression	49.99%	50.00%	0.01%
$t_{53}$	AT2R mediated PGI2 production by endothelial cells	46.79%	49.44%	2.65%
$t_{54}$	synthesis of PGI2 by endothelial cells	50.00%	50.01%	0.00%
$t_{55}$	lowering blood pressure by PGI2	48.32%	49.42%	1.10%
$t_{56}$	the immune system activation in interstitium under local hypertonic state	25.00%	25.00%	0.00%
$t_{57}$	TonEBP synthesis under local hypertonic state	24.99%	24.99%	0.00%
$t_{58}$	VEGF-C activation	24.98%	24.98%	0.00%
$t_{59}$	VEGF-C with VEGFR3 binding	18.28%	17.48%	−0.80%
$t_{60}$	modification of lymph capillary network via VEGFR3	18.27%	17.48%	−0.80%
$t_{61}$	process in lymphatic endothelium	50.00%	50.00%	−0.01%
$t_{62}$	VEGF-C with VEGFR2 binding (canonical signaling)	7.32%	7.50%	0.18%
$t_{63}$	eNOS synthesis induction calcium and phosphorylation dependent	7.32%	7.50%	0.18%
$t_{64}$	NO increase	7.32%	7.50%	0.18%
$t_{65}$	muscles relaxation	7.32%	7.49%	0.18%
$t_{66}$	health	27.80%	28.46%	0.65%
$t_{67}$	NADPH oxidase activation via VEGFR2	25.95%	27.68%	1.72%
$t_{68}$	vascular endothelium VEGFR2 expression	50.01%	50.00%	−0.01%
$t_{69}$	VEGF pathway (ligand independent, non canonical signaling)	16.73%	14.81%	−1.91%
$t_{70}$	peroxynitrire (ONOO anion) formation	35.16%	30.04%	−5.11%
$t_{71}$	oxidation of BH4 (eNOS cofactor)	35.14%	30.03%	−5.11%
$t_{72}$	superoxide anion synthesis by eNOS uncoupling	23.68%	15.02%	−8.67%
$t_{73}$	low NO synthesis	23.67%	15.01%	−8.65%

**Table 7:** The list of places that will not carry any tokens during simulation as the effect of the $m_{5}$ knockout.

Place	Biological Meaning
$p_{0}$	angiotensinogen
$p_{1}$	angiotensin I
$p_{2}$	high angiotensin II
$p_{4}$	BR2 in EC
$p_{5}$	TNF alpha
$p_{7}$	ET1
$p_{8}$	C-reactive protein (CRP)
$p_{23}$	Th lymphocytes
$p_{26}$	T lymphocytes in adventitia and perivascular adipose tissue
$p_{27}$	bradykinin and BR1 complex
$p_{28}$	BR1
$p_{29}$	bradykinin
$p_{32}$	kalikrein
$p_{33}$	XII factor
$p_{34}$	HMWK with zinc
$p_{35}$	damaged endothelium with platelet aggregation
$p_{39}$	TNF alpha-TNFR1 complex

**Table 8:** The impact of each MCT set knockout on the net according to the simulation knockout.

MCT Set Knocked out	MCT Sets Whose Transitions Are Inactive
	According to the Simulation Knockout
$m_{1}$	-
$m_{2}$	$m_{1}$, $m_{3}$, $m_{8}$
$m_{3}$	$m_{1}$, $m_{8}$
$m_{4}$	-
$m_{5}$	$m_{1}$, $m_{2}$, $m_{3}$, $m_{8}$
$m_{6}$	-
$m_{7}$	-
$m_{8}$	-
$m_{9}$	$m_{1}$, $m_{2}$, $m_{3}$, $m_{8}$
$m_{10}$	-

## Abbreviations

The following abbreviations are used in this manuscript:

ACE	angiotensin-converting enzyme
ACEI	angiotensin converting enzyme inhibitors
ADMA	asymmetric dimethylarginine
APC	antigen-presenting cell
AT1R	angiotensin type 1 receptor
AT2R	angiotensin type 2 receptor
BH4	tetrahydrobiopterin
BR1	bradykinin receptor type 1
CCR5	receptor for RANTES
CRP	C-reactive protein
eNOS	endothelial nitric oxide synthase
ET1	endothelin 1
HMWK	high-molecular-weight kininogen
IL-17	interleukin 17
MCT sets	Maximal Common Transition sets
MPS	mononuclear phagocyte system
MSS	Mean Split Silhouette
NO	nitric oxide
PGE2	prostaglandin
PGI2	prostacyclin
RANTES	regulated on activation, normal T-cell expressed and secreted
RAS, RAA	renin-angiotensin system
ROS	reactive oxygen species
TNF alpha	tumor necrosis factor alpha
TonEBP	tonicity enhancer binding protein
UPGMA	Unweighted Pair Group Method with Arithmetic Mean
VEGF-C	vascular endothelial growth factor C
VEGFR2	vascular endothelial growth factor receptor 2
VEGFR3	vascular endothelial growth factor receptor 3
